# Reduced serum interleukin-2 associates with higher motor severity and along with CD4 T cell alterations may be an early event in isolated REM Sleep Behaviour Disorder

**DOI:** 10.1016/j.bbih.2026.101257

**Published:** 2026-05-12

**Authors:** Fatima Afaar, Priscilla Youssef, Laura Hughes, Michelle Chua, Elie Matar, Woojin S. Kim, Glenda M. Halliday, Simon J.G. Lewis, Nicolas Dzamko

**Affiliations:** a School of Medical Sciences, Faculty of Medicine and Health and the Brain and Mind Centre, University of Sydney, Camperdown, NSW, 2050, Australia; b Sydney Medical School, Faculty of Medicine and Health, Brain and Mind Centre, University of Sydney, Department of Neurology, Royal Prince Alfred Hospital, Camperdown, NSW, 2050, Australia; c Parkinson's Disease Research Clinic, Macquarie Medical School, Macquarie University, 75 Talavera Road, NSW, Australia

## Abstract

**Background:**

Emerging evidence indicates that peripheral immune changes occur in patients with isolated REM sleep behaviour disorder (iRBD) and may contribute to the conversion of this dream enactment disorder to a neurodegenerative synucleinopathy such as Parkinson's disease, dementia with Lewy bodies or multiple system atrophy. However, with only a limited number of studies conducted, the extent to which immune changes occur across diverse iRBD cohorts remains to be determined.

**Objectives:**

We therefore aimed to assess peripheral immune changes in an Australian cohort of iRBD patients (n = 65) compared to controls (n = 35).

**Methods:**

A 9-plex cytokine assay was used to measure serum levels of IFNγ, IL-10, IL-12, IL-17A, IL-1β, IL-2, IL-4, IL-6 and TFNα. Flow cytometry was used to assess T cell populations in the same participants.

**Results:**

Exploratory analyses revealed lower levels of the T cell regulating cytokine interleukin 2 (IL-2) in participants with iRBD, along with lower frequencies of CD4 T cells positive for IL-2, IL-4 and IL-10.

**Conclusions:**

These results add to evidence of immune alterations in iRBD and suggest that dysregulation of cytokines with anti-inflammatory properties may be an early event that could associate with subsequent development of a neurodegenerative synucleinopathy.

## 1 Introduction

Isolated rapid eye movement sleep behaviour disorder (iRBD) is a parasomnia resulting in a loss of atonia and dream enactment (Roguski et al., 2020). It is now recognised that over 70% of people diagnosed with iRBD are at risk of developing a neurodegenerative synucleinopathy, a disease associated with pathological aggregation of alpha-synuclein protein in the brain, within 5-15 years of diagnosis (Postuma et al., 2019a). Synucleinopathies include Parkinson's disease (PD), dementia with Lewy Bodies (DLB) and multiple system atrophy (MSA) (Galbiati et al., 2019). These conditions are characterised by variable degrees of motor and non-motor symptoms that are associated with selective patterns of neuronal loss across dopaminergic, cholinergic, noradrenergic and serotonergic neurons (Marti et al., 2003). At the time of clinical diagnosis, up to 50% of dopamine neurons may have degenerated, with clinical extrapolations suggesting that PD may begin 10-20 years prior with a long prodromal phase (Kalia et al., 2015). Thus, although not all people with iRBD will develop PD, the study of such patients may provide insight into the earlier prodromal stages of PD and related synucleinopathies, which is critical for improving both diagnostics and therapeutic interventions (Miglis et al., 2021).

The exact causes driving synucleinopathies remain to be determined, however both the central and peripheral immune systems have been heavily implicated in pathogenesis (Weiss et al., 2022; Harms et al., 2023; Roodveldt et al., 2024). In particular, common PD risk genes such as leucine rich repeat kinase 2 (*LRRK2*) and glucocerebrosidase (*GBA1*) are involved in immune signaling pathways (Dzamko et al., 2015) and many studies have measured altered cytokine levels in PD patients (Dzamko, 2023). Peripheral mononuclear cell populations are also altered in PD, in particular many studies have measured altered populations of T lymphocytes (Contaldi et al., 2022). The extent and direction of changes in cytokines and immune cell populations differ across studies and can be influenced by many factors. Age, sex, immune-related comorbidities, medications, genetic status and lifestyle factors can all modulate the immune system (Munoz-Delgado et al., 2025). Assay methodology and statistical approaches to measure cytokines can also provide different outcomes (McKinski et al., 2025). Despite the challenges however, meta-analysis across 152 studies reported an increase in peripheral proinflammatory cytokines including interleukin (IL)-6, tumor necrosis factor alpha (TNFα), IL-1β and c-reactive protein (CRP) with PD (Qu et al., 2023). Meta analysis of lymphocyte studies has also been performed, with 21 studies containing almost 1000 PD patients demonstrating a reduction in CD3^+^ and CD4^+^ T cells with PD (Jiang et al., 2017).

Neuroimaging has demonstrated an upregulation of microglial activity in iRBD (Stokholm et al., 2017) and this has been correlated with subsequent dopaminergic loss (Staer et al., 2024). Thus, neuroinflammation may be an early event contributing the development of synucleinopathies. An upregulation of peripheral inflammatory cytokines, particularly interleukin IL-6, IL-8, IL-10 and TNFα, has also been reported in patients with iRBD (Kim et al., 2019, 2020; Zhang et al., 2020; Li et al., 2022; Huxford et al., 2025), suggesting that altered peripheral immunity may also be an early feature of subsequent conversion to synucleinopathy. Although it remains to be determined exactly how peripheral immunity contributes to synucleinopathy, crosstalk between the central and peripheral immune systems is recognised, with peripheral immunity directly affecting blood brain barrier integrity, glial cell activation and the infiltration of lymphocytes and monocytes into the brain (Roodveldt et al., 2024; Tumpa et al., 2025). Changes in both monocyte (Farmen et al., 2021) and lymphocyte (Zheng et al., 2024) subsets have also been demonstrated in patients with iRBD. In regard to lymphocytes, alterations in both CD4 and CD8 T cell subsets have been reported (Zheng et al., 2024), along with changes in transcription factors that mediate T cell fate (De Francesco et al., 2021). However, the study of peripheral immunity in iRBD remains limited with a need for further validation across diverse cohorts. Moreover, cytokines and lymphocyte populations have largely been studied independently, and therefore the extent of any association between these biological immune effectors remains unclear. Therefore, to further build on evidence for immune dysfunction in iRBD we performed an exploratory study to assess a panel of cytokines and T cell populations in an Australian cohort to test our hypothesis that pro-inflammatory cytokines would be increased with iRBD.

## 2 Methods

### 2.1 Participant details

Ethical approval for this study was obtained from the University of Sydney Human Research Ethics Committee (#2017/826). Participants with polysomnography confirmed iRBD and neurologically normal control subjects without a diagnosed neurological or psychiatric disease (predominantly spousal controls) were recruited via the Parkinson's disease research clinic at the Brain and Mind Centre, University of Sydney. Participants underwent clinical assessment and were screened for the presence of comorbid chronic inflammatory, autoimmune, infectious, or metabolic diseases that might independently modulate cytokine production and were excluded if present. All recruited participants provided written informed consent. Participants were assessed on the Movement Disorders Unified Parkinson's Disease Rating Scale part three (MDS-UPDRS-III) for parkinsonian features and the Montreal Cognitive Assessment (MoCA) as a measure of global cognition. Impaired olfaction and colour discrimination vision have been highlighted as risk factors for transitioning to synucleinopathy (Postuma et al., 2019b), and as such these were assessed with Sniffin’ Sticks (Kobal et al., 1996) and the Farnsworth-Munsell 100 hue test (Farnsworth, 1943), respectively. Subjective iRBD was measured with the RBD Screening Questionnaire (Stiasny-Kolster et al., 2007) and symptom duration since diagnosis was recorded at the time of blood collection. Sometimes participants were unable to perform all clinical assessments resulting in some missing data. Demographic and clinical data are provided in Table 1. Information on current prescribed anti-inflammatory medication use was also recorded and is provided in Supplementary Table 1, with 7 control and 17 iRBD participants having anti-inflammatory medication use, with the most common being daily use of aspirin. All blood draws were performed under comparable conditions by an experienced phlebotomist at the same location. Blood draws occurred mostly in the morning with 64% of samples collected between the hours of 9am-11am. The remainder blood draws were performed in the early afternoon, predominantly between the hours of 12pm-2pm. Participants were not fasted.

**Table 1:** **Demographic and clinical data of study participants.** Data shows mean ± standard error. Significance was determined at *p* value < 0.05 using an independent samples T-test. ∗Indicates *p* < 0.05. FM-100 Hue, Farnsworth Munsell 100 Hue test; MDS-UPDRS-III, MDS Unified Parkinson's Disease Rating Scale Part III; MMSE, Mini-Mental State Examination; MoCA, Montreal Cognitive Assessment; RBDSQ, REM sleep behaviour disorder screening questionnaire; % anti-inflammatory is the percentage of the group with a prescription for anti-inflammatory medication.

	Control	iRBD	*p* value
Sample size	35	65	
Age (years)	67 ± 1	68 ± 1	0.672
Sex (%M)	57%	75%	0.060
Years since diagnosis	NA	2.6 ± 0.5	
MDS-UPDRS-III	5.0 ± 0.9	7.6 ± 0.9	0.085
Sniffin Sticks	9.8 ± 0.3	7.5 ± 0.4	<0.001∗
FM-100 Hue	93.8 ± 7.6	101.8 ± 7.6	0.533
RBDSQ	2.4 ± 0.5	7.9 ± 0.5	<0.001∗
MMSE	28.4 ± 0.3	28.3 ± 0.2	0.830
MoCA	26.8 ± 0.5	26.7 ± 0.03	0.752
% Using Anti-inflammatory drugs	20%	26%	0.492
CD3^+^ T cell viability	81 ± 1.3%	82 ± 1.1%	0.511

### 2.2 Serum collection

Serum from each participant was collected in 8.5 mL Serum Separator Tubes (SST) II Advanced tubes (BD Biosciences). Serum samples were incubated at room temperature for 30 min to allow blood to clot prior to centrifugation at 1500×*g* for 15 min at 21 °C (acceleration at 9 and deceleration at 5 using a Thermo Scientific Multifuge X1 Centrifuge). Serum was then transferred into a 15 mL falcon, mixed by inversion and then partitioned into 500 μL aliquots, and immediately placed in −80 °C until required for experiments.

### 2.3 Serum inflammatory cytokine measurement

Meso Scale Discovery (MSD) S-Plex Proinflammatory Kits (#K15396S) were used to simultaneously measure nine serum inflammatory cytokine markers (IFNγ, IL-10, IL-12, IL-17A, IL-1β, IL-2, IL-4, IL-6 and TFNα). Serum samples were thawed, centrifuged at 2000×*g* for 3 min at 4 °C and kept on ice while assay reagents were bought to room temperature. 25 μL of undiluted serum sample was added per well, and the assay performed as per manufacturer's instructions. Plates were read on the MESO QuickPlex SQ 120 MM plate reader (MSD). The calibration curves used to calculate the serum cytokine concentrations were established by fitting the signals from the calibrators using a 4-parameter logistic model with a 1/Y 2 weighting. Data were obtained and analysed using the MSD discovery workbench software. All calibrators and samples were performed in duplicates with average values used for the final statistical analysis. A small number of cytokines below the limit of detection (IL1β n = 6, IL-2 n = 2 and IL-6 n = 2) were substituted with a nominal value equal to half of the lowest standard curve concentration for that particular analyte to reflect that these values were very low.

### 2.4 Peripheral blood mononuclear cell (PBMC) collection

Up to 18 ml of whole blood was collected by venepuncture into sodium heparin tubes (BD Biosciences). PBMCs were isolated from whole blood following the SepMate manufacturer instructions (STEMCELL Technologies). Specifically, whole blood was gently mixed with an equal volume of phosphate-buffered saline (PBS, Thermo Fisher) containing 2% heat inactivated low endotoxin qualified fetal bovine serum (FBS, Thermo Fisher) in a 50 mL tube. Diluted blood was then carefully transferred to a 50 mL SepMate tube (STEMCELL Technologies) containing 17 mL of density gradient medium. Samples were centrifuged at 1200×*g* for 10 min at 21 °C (acceleration at 9 and deceleration at 9 using a Thermo Scientific Multifuge X1 Centrifuge) and the enriched PBMC layer transferred to a new 50 mL tube. PBMCs were then washed with PBS containing 2% FBS and centrifuged at 300×*g* for 8 min at 21 °C (acceleration at 9 and deceleration at 9). Wash buffer was discarded, and cells were resuspended in 10 mL of PBS containing 2% FBS, and the cell count was determined using 0.4% trypan blue stain (Thermo Fisher) and a Countess automated cell counter (Thermo Fisher). A final centrifugation at 300×*g* for 8 min at 21 °C (acceleration at 9 and deceleration at 9) was then performed. PBMCs were resuspended in cryopreservation media comprised of RPMI 1640 (Thermo Fisher), 20% FBS and 10% dimethyl sulfoxide (DMSO, Sigma-Aldrich), in cryovials at a density of 4 × 10^6^ cells/mL and then placed in a MrFrosty Freezing Container (Thermo Fisher) containing isopropanol (Sigma-Aldrich) overnight in a −80 °C freezer. The following morning PBMC samples were transferred to a vapour phase liquid nitrogen tank until required for experiments.

### 2.5 Flow cytometry measurement of T-cell subsets and intracellular cytokines

PBMC cryovials were rapidly thawed in a 37 °C water bath until a small piece of ice remained, added to warmed (37 °C) culture media (RPMI 1640 supplemented with 10% v/v FBS, 50 U/mL penicillin/streptomycin and 2 mM L-glutamine (all from Thermo Fisher) and centrifuged at 300×*g* for 10 min at room temperature (RT). Supernatant was removed and PBMCs resuspended in culture media for cell counting using Trypan blue (Thermo Fisher) and an automated cell counter (Countess II-FL, Life Technologies). PBMCs were diluted to 1 × 10^6^ per mL in culture media and 1 × 10^6^ cells plated per well in 24-well non tissue culture treated plates (Sigma-Aldrich) and treated with GolgiPlug (BD Biosciences) at 1:1000 ± 50 ng/mL phorbol 12-myristate 13-acetate (PMA, Sigma-Aldrich) and 1 μg/mL ionomycin (Sigma-Aldrich) and incubated at 37 °C, 5% CO_2_ for 4 h. Cells were then transferred into a 96-well U-bottom plate (Sigma-Aldrich) for immunofluorescent staining. All centrifugation steps were performed at 300×*g* for 5 min at 4 °C. Cells were centrifuged and resuspended in 50 μL per sample of PBS containing Fc receptor blocking solution, viability stain, and antibodies directed to cell surface markers at dilutions specified in Supplementary Table 2 and incubated at 4 °C for 30 min, shielded from light. Samples were washed in fluorescence-activated cell sorting (FACS) buffer, PBS with 1% FBS, 1 mM ethylenediaminetetraacetic acid disodium salt dihydrate (EDTA, Sigma-Aldrich) and 25 mM HEPES (Sigma-Aldrich) and resuspended in 100 μL BD Phosflow™ Fix Buffer I (BD Biosciences) and incubated for 20 min at RT. Samples were washed in FACS buffer and resuspended in 100 μL of 1 x Perm/Wash buffer (BD Biosciences) containing antibodies directed to intracellular markers at dilutions specified in Supplementary Table 2 and incubated at 4 °C for 45 min. Cells were washed twice 1 x Perm/Wash buffer and resuspended in 100 μL BD Phosflow™ Fix Buffer I (BD Biosciences) and incubated for 10 min at RT to final-fix cells. Cells were washed in FACS buffer and resuspended in 250 μL FACS buffer for sample acquisition. Data were acquired on a Cytek Aurora Spectral Analyser using SpectroFlo software, with a minimum of 100,000 events were collected per sample in most instances. Flow Cytometry Standard (FCS) files were then imported into FlowJo version 10.10 software (BD Biosciences) for analysis. An example of the gating strategy employed is shown in Supplementary Fig. 1.

### 2.6 Statistical analysis

Data analysis was performed using SPSS version 26 (IBM). Raw cytokine data was not normally distributed with all cytokines having p < 0.001 using the Kolmogorov-Smirnov test. Cytokines were therefore log transformed to improve normal distribution. Following log transformation, all cytokines except interferon gamma (IFNγ) were normally distributed using the Kolmogorov-Smirnov test. After log transformation IFNγ had a p-value of 0.09 and retained a right skew. Parametric tests were still used for IFNγ despite the residual skew to include the use of covariates. Differences between groups for clinical data were determined using independent samples T-tests, with the exception of sex and anti-inflammatory medication, for which a Pearson's Chi Square test was used. For the analysis of anti-inflammatory medication use, participants were given a nominal value of 0 or 1 for yes or no respectively for current anti-inflammatory medication use. Differences between groups for cytokine data were determined using general linear model univariate analyses covarying for age, sex and the use of anti-inflammatory medication. Sample size calculations were not performed. Homogeneity of regression slopes was assessed by testing the interaction between the grouping variable and covariates within each general linear model. For all models, a non-significant interaction effect (p > 0.05) was observed indicating that the assumption of parallel regression slopes across groups was met. For general linear models, the estimated marginal means for cytokine variables were presented after adjustment for age, sex and anti-inflammatory medication use. Pearson correlations were used to determine any associations between clinical variables and log transformed cytokine levels. For correlation analyses a Bonferroni adjusted p-value was applied to account for multiple comparisons. For all other tests, significance was accepted a p < 0.05 and should be considered exploratory. Graphs were generated using Prism version 10 (GraphPad).

## 3 Data sharing

The data that support the findings of this study are available from the corresponding author upon reasonable request.

## 4 Results

### 4.1 Cohort characteristics

Analysis of the demographic data showed that participant groups were matched for age. The proportion of males in the iRBD group trended higher than controls but was not significantly different (p = 0.06) (Table 1). As expected, iRBD participants scored higher on the RBD-Q and performed significantly worse on the Sniffin’ Sticks smell test (both p < 0.001) but did not differ in their colour discrimination vision compared to controls (Table 1). The iRBD participants trended to have a higher score on the UPDRS-III scale, but this was not statistically significant (p = 0.085). One iRBD participant was recorded as receiving dopamine replacement medication that was administered for Restless Legs Syndrome. The groups did not differ on cognitive function as assessed by MoCA and MMSE (both p > 0.05) (Table 1). The proportion of participants prescribed anti-inflammatory medication also did not differ between the two groups (p = 0.492) (Table 1).

### 4.2 Serum Interleukin-2 is significantly reduced in isolated REM sleep behaviour disorder

Univariate analysis of the cytokine data, covarying for age, sex and the use of anti-inflammatory medication showed a significant reduction in the serum levels of IL-2 in the iRBD group (12% reduction, p = 0.001, Cohen's d = 0.75) (Table 2). No other measured cytokines were significantly different between the two groups. Regarding the covariates, age was a significant factor in the assessment of IL-10 (p = 0.015), IL-2 (p = 0.041) and IL-6 (p = 0.031), whereas sex was a significant factor in the assessment of IL-10 (p = 0.008). The use of anti-inflammatory medication was not a significant factor for any of the measured cytokines.

**Table 2:** **Cytokine data of study participants.** A Mesoscale discovery 9-plex cytokine assay was used to measure nine representative cytokines in serum from control (n = 35) and iRBD (n = 65) participants. The table shows the estimated mean ± standard error of log transformed cytokine levels after univariate analysis covarying for age, sex and the use of anti-inflammatory medication. Significance was determined at *p* value < 0.05, and is indicated with ∗.

	Control	iRBD	*p* value
IFNγ	2.687 ± 0.05	2.681 ± 0.04	0.926
IL-10	2.823 ± 0.05	2.744 ± 0.04	0.225
IL-12	2.532 ± 0.06	2.528 ± 0.04	0.954
IL-17A	2.582 ± 0.07	2.490 ± 0.05	0.272
IL-1β	1.724 ± 0.07	1.696 ± 0.05	0.745
IL-2	2.071 ± 0.06	1.816 ± 0.04	0.001∗
IL-4	1.648 ± 0.07	1.622 ± 0.05	0.749
IL-6	3.318 ± 0.04	3.292 ± 0.03	0.673
TNFα	2.996 ± 0.03	2.966 ± 0.02	0.403

### 4.3 Serum interleukin 2 negatively correlates to UPDRS-III

**Fig. 1:**
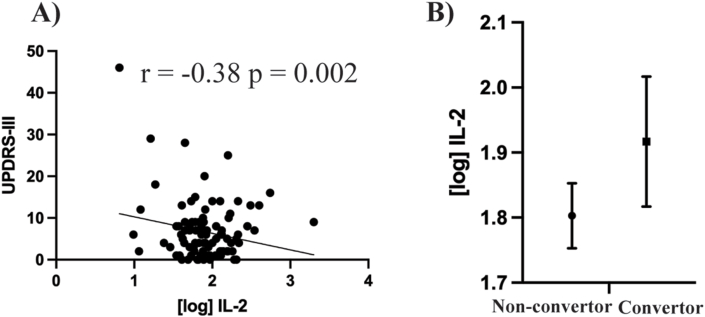
**IL-2 correlates with clinical data.** Serum levels of IL-2 negatively correlated with the MDS-UPDRS-III disease severity scale in participants with iRBD (n = 65) (**A**). Pearson correlations were performed with dots on the graphs indicating individual data points. **B**) Serum levels of IL-2 were compared between iRBD patients that had subsequently converted (n = 13) or not converted (n = 52) to a diagnosis of synucleinopathy at the time of writing. The graphs show the estimated marginal mean and the standard error of the mean following univariate analysis covarying for age, sex and the use of anti-inflammatory medication.

Correlation analysis was performed to determine if serum levels of IL-2 in the iRBD group were associated with available clinical data. This showed a significant negative correlation between serum IL-2 levels and the MDS-UPDRS-III motor severity scale (Pearson correlation coefficient r = −0.38, p = 0.002) (Fig. 1A). There were no significant correlations with the other clinical variables (Supplementary Table 3). Thirteen of the 65 iRBD participants originally recruited into this study had phenoconverted to a synucleinopathy at the time of writing (9 x PD, 3 x DLB, 1 x MSA). Univariate analysis, covarying for age, sex and the use of anti-inflammatory medication revealed that baseline serum levels of IL-2 were not different between those that had converted to a synucleinopathy and those that did not (p = 0.314) (Fig. 1B). None of the covariates had a significant effect on the analysis (all > p = 0.05) suggesting that although lower levels of IL-2 may be associated with higher motor severity scores, serum levels of IL-2 may not necessarily be predictive of near-future conversion of iRBD to a synucleinopathy. However, this outcome should be interpreted with caution due to the low sample size, limited power and a non-longitudinal design for prediction.

### 4.4 Reduced interleukin-2, 4 and 10 positive T-cells with isolated REM sleep behaviour disorder

**Fig. 2:**
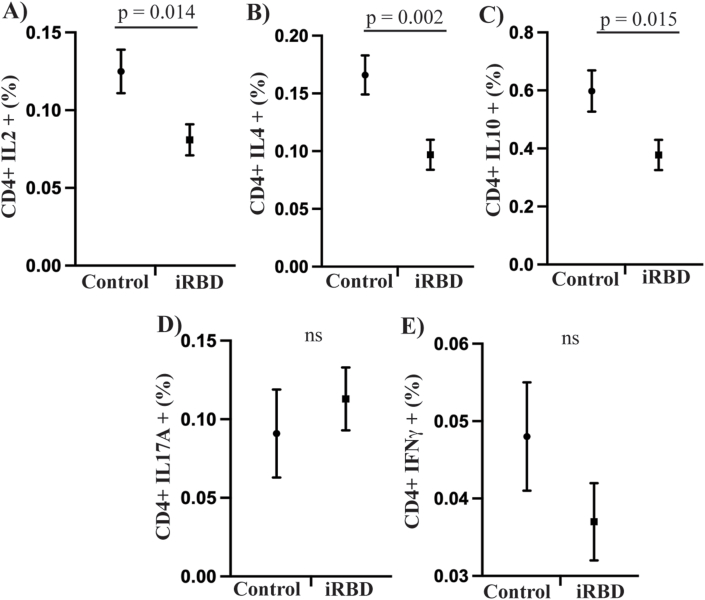
**Altered CD4 T cell frequencies with iRBD.** Flow cytometry was used to assess the resting frequencies of CD4 T cells positive for IL-2 (**A**), IL-4 (**B**), IL-10 (**C**), IL-17A (**D**) and IFNγ (**E**). The graphs show the estimated marginal mean and the standard error of the mean following univariate analysis covarying for age and sex.

IL-2 is predominantly secreted by naïve T cells and helper Th1 cells to induce the proliferation of regulatory T cells (Treg). We therefore examined the populations of these cells across the groups, both in a resting state and following activation with PMA and ionomycin. Under resting conditions there was no difference in the percentages of CD4, CD8, Th1 (CD4^+^ TBET+) or Treg (CD4^+^ FoxP3+) populations between the two groups following univariate analyses covarying for age and sex (Supplementary Table 4). However, in the iRBD group the percentage of CD4 T cells that were positive for IL-2 (35% decrease, p = 0.014, Cohen's d = 0.57, Fig. 2A), IL-4 (42% decrease, p = 0.002, Cohen's d = 0.73, Fig. 2B) and IL-10 (37% decrease, p = 0.015, Cohen's d = 0.58, Fig. 2C) was significantly less than in controls. The only significant covariate in the analysis of these T cell subsets was sex, which was a significant factor in the analysis of IL-10 positive T cells (p = 0.012). CD4 T cells that were positive for IL-17A (Fig. 2D) and IFNγ (Fig. 2E) did not differ between the two groups (both p > 0.05). There was no correlation of IL-2, IL-4 or IL-10 positive T cell subsets to either UPDRS-III or serum levels of IL-2 (all p > 0.05) (Supplementary Table 5). Activation of T cells resulted in marked upregulation of CD4 T cells positive for IL-2, IL-4, IL-17A and IFNγ, but there were no longer any differences between groups (Supplementary Fig. 2A–E). The percentage of Treg cells was also increased following activation but again was not different between iRBD and controls (Supplementary Fig. 2F).

## 5 Discussion

This study aimed to determine if peripheral cytokine levels were altered in an Australian cohort of participants with iRBD compared to controls. We found that serum levels of IL-2 were significantly reduced in iRBD participants, as was the frequency of CD4 T cells expressing IL-2. Our findings of reduced IL-2 in iRBD differ from the results of Kim et al., who found no difference between control and iRBD in serum levels of IL-2 (Kim et al., 2020). Differences between these studies were that that current study had more than double the sample size; employed the use of covariates in statistical analysis; and the study cohort had a shorter time duration following their iRBD diagnosis. The current study also used a more sensitive Mesoscale Discovery S-plex inflammatory cytokine panel. This may be an important factor as IL-2 has also been assessed in plasma from iRBD patients and controls but was reported as below detection (Zhang et al., 2020) or at very low detection (Kim et al., 2019), with both studies using the less sensitive Mesoscale V-plex pro-inflammatory panel. Thus, the current study may comprise a more accurate evaluation of low abundance cytokines such as IL-2. Our results also differ from other studies that have found elevated levels of TNFα in both iRBD serum and plasma (Kim et al., 2019, 2020; Huxford et al., 2025), whereas the current study detected no significant difference in the levels of this cytokine. This may be methodological since different assay kits were used, or possibly reductions in cytokines with anti-inflammatory properties such as IL-2 may precede and contribute to subsequent increases in pro-inflammatory cytokines such as TNFα. In the current study, the time since diagnosis of iRBD was an average of 2 years, which is much shorter than within the other reported cohorts above. Additionally, TNFα has been linked to phenoconversion (Kim et al., 2020; Huxford et al., 2025) and since the current study cohort was early in iRBD duration, only a limited number of participants (20%) had converted to a synucleinopathy at the time of writing. Since TNFα was not significantly increased in this study, it may be possible that most of our cohort was sampled prior to the development of low-grade inflammation. Additional studies, including iRBD cohorts with greater disease stage heterogeneity and/or longitudinal validation are still required to test the hypothesis that an early loss of immunoregulatory cytokines precedes later pro-inflammatory elevations.

A common source of plasma IL-2 is secretion from activated CD4 T cells, where it functions to modulate the immune response by maintaining the balance of Th1 and Th2 cells, and in particular the proliferation of Treg cells (Goleij et al., 2025). In addition to reduced plasma levels of IL-2, this study also uncovered a reduction in the frequency of CD4^+^ IL2+ T cells, indicating less IL-2 producing T cells in iRBD patients under resting conditions. Although a correlation was not seen between the levels of these T cells and serum IL-2, suggesting a more complex relationship. The frequency of anti-inflammatory producing IL-10 and IL-4 CD4 T cells at rest was also reduced in iRBD patients. When T cells were activated to induce cytokine production, no differences remained between control and iRBD groups suggesting that T cells may be functionally normal. However, it is noteworthy that a reduction in CD4^+^ IL-4+ T cells in iRBD has been reported previously, following stimulation of PBMCs with phorbol myristate acetate and ionomycin (Zheng et al., 2024). Both the concentration of ionomycin and the time point of analysis following activation differed between our study and the latter. Thus, it is plausible that the detection of differences following stimulation are dependent on the conditions employed. For example, at least two other studies have demonstrated impaired IL-2 production from PD patient PBMCs using different treatment paradigms (Bessler et al., 1999; Kluter et al., 1995). Thus, a reduction in T cells secreting cytokines with anti-inflammatory properties could over time affect immune homeostasis.

Despite reduced IL-2 levels, both the current study and the study of Zheng et al. (2024), did not find a difference in the baseline or stimulated frequencies of FoxP3+ Treg cells. Intriguingly, several studies have demonstrated a reduced population of Treg cells in patients with PD (Baba et al., 2005; Kustrimovic et al., 2018; Alvarez-et al., 2019). Discrepancies can result from different markers being used to the define Treg populations. In the current study Treg cells were generically defined as CD4+FoxP3+, which does not fully capture the functional or phenotypic heterogeneity relevant to immunoregulation. Additional markers could be incorporated into replication studies to determine if distinct subsets of circulating Tregs such as naive versus activated are particularly effected. However, daily low dose intraperitoneal injection of recombinant IL-2 increased Treg frequency and protected against neurodegeneration in toxin induced PD mouse model (Markovic et al., 2022). In the current study IL-2 also inversely correlated to the UPDRS-III motor severity score and reduced IL-2 has previously been inversely corelated to UPDRS and cognitive impairment in patients with DLB (King et al., 2018). Although further longitudinal studies investigating peripheral and central immune cross talk are needed for validation, this may suggest that defective IL-2 production is an early event contributing to the development of neurodegeneration through an ongoing inability to suppress immune responses, resulting in glial activation and neuroinflammation.

This study also has limitations to acknowledge. Similar to most other studies on inflammation in iRBD, this study comprised a single cross-sectional cohort and the lack of a validation cohort remains a limitation. Although this study employed anti-inflammatory medication as a covariate, it remains unknown whether changes in IL-2 or T cell populations are transient and/or how changes continue to develop longitudinally. Moreover, the approach employed to use anti-inflammatory medication as a covariate does not consider specific doses or classes of drugs. Additionally, only a limited number of iRBD patients were confirmed as converting to synucleinopathy and some participants in this study may never develop a neurodegenerative disease. The extent to which immune changes in iRBD relate to the development of different synucleinopathies also remains to be determined. Changes in T cell populations at baseline were also small, and whether they are functionally relevant under physiological activation conditions remains to be determined. We also did not assess total leukocyte or lymphocyte counts in the cohort and therefore can't rule out that differences in T cell subset frequencies may be reflective of lower levels of immune cells in general. Dietary supplements, fasting status, the time of blood draw or other lifestyle factors that may modulate inflammation were not formally assessed as part of this study and could impact upon results.

In summary the data obtained support the observation that peripheral immune changes occur in iRBD. Possibly these changes may associate with the subsequent development of PD and other synucleinopathies.

## Funding sources

This work was funded by a bequest from Francis Patrick Gill and the Australian Parkinson's Mission, which was conceived as an Australian-led collaboration between the Garvan Institute of Medical Research, The University of Sydney, The Cure Parkinson's Trust (UK), The Michael J Fox Foundation (USA) and the Shake It Up Australia Foundation and Parkinson's Australia.

## CRediT authorship contribution statement

**Fatima Afaar:** Data curation, Formal analysis, Writing – original draft. **Priscilla Youssef:** Investigation, Methodology, Supervision, Writing – original draft. **Laura Hughes:** Formal analysis, Investigation, Methodology, Writing – original draft. **Michelle Chua:** Investigation, Methodology, Writing – original draft. **Elie Matar:** Data curation, Investigation, Writing – review & editing. **Woojin S. Kim:** Resources, Supervision, Writing – review & editing. **Glenda M. Halliday:** Project administration, Resources, Supervision, Writing – review & editing. **Simon J.G. Lewis:** Data curation, Investigation, Methodology, Resources, Supervision, Writing – original draft, Writing – review & editing. **Nicolas Dzamko:** Conceptualization, Data curation, Formal analysis, Funding acquisition, Investigation, Methodology, Project administration, Resources, Supervision, Visualization, Writing – original draft, Writing – review & editing.

## Declaration of competing interest

The authors have no competing interests to declare.

## Data Availability

Data will be made available on request.
