# Supplementary material for: Reduced serum interleukin-2 associates with higher motor severity and along with CD4 T cell alterations may be an early event in isolated REM Sleep Behaviour Disorder
Source: Brain Behav Immun Health. 2026 May 12;54:101257. doi: 10.1016/j.bbih.2026.101257 (PMC13195789; doi:10.1016/j.bbih.2026.101257)
Supplement: Multimedia component 1 [file mmc1.pdf]

Medication	Number of iRBD participants using	Dose	Number of control participants using	Dose
Aspirin	13	100 mg	4	100 mg
Ibuprofen	2	400 mg	2	400 mg
Naproxen	0		1	1000 mg
Celecoxib	1	200 mg	0	
Diclofenac	1	50 mg	0	

Supplementary Table 1. Current anti-inflammatory medication use. Table shows anti-inflammatory medications being taken by participants in this study.

Specificity	Fluorophore	Manufacturer	Catalogue #	Titrated to
FcR		BioLegend	422302	1:20
Live/Dead		BD Biosciences	564997	1:1000
CD3	APC	BD Biosciences	570820	1:20
CD4	BV711	BD Biosciences	563028	1:20
CD8	AF700	BD Biosciences	570799	1:20
T-Bet	BV421	BD Biosciences	563318	1:20
Foxp3	AF488	BD Biosciences	561181	1:20
IL-2	PE	BD Biosciences	569370	1:20
IL-10	PE-Cy7	BD Biosciences	567407	1:20
IFN-γ	AF647	BD Biosciences	563495	1:20
IL-4	RB780	BD Biosciences	569087	1:20
IL-17A	RY610	BD Biosciences	571173	1:20

Supplementary Table 2. Antibodies employed in this study. Table shows reagents and antibodies used for flow cytometry in this study.

Correlations

		LogIL2	MDS UPDRSIII	Years since diagnosis	Age	MMSE	MoCA	RBDSQ	Hue test	Sniffin sticks
LogIL2	Coefficient	--								
	N	65								
MDS UPDRSIII	Coefficient	-.375**	--							
	Sig. (2-tailed)	.002								
	N	63	63							
Years since diagnosis	Coefficient	-.012	.357**	--						
	Sig. (2-tailed)	.924	.004							
	N	64	62	64						
Age	Coefficient	.126	.302*	.304*	--					
	Sig. (2-tailed)	.319	.016	.014						
	N	65	63	64	65					
MMSE	Coefficient	-.081	.096	.203	-.003	--				
	Sig. (2-tailed)	.549	.477	.133	.984					
	N	57	57	56	57	57				
MoCA	Coefficient	.086	-.274*	-.171	-.128	.268*	--			
	Sig. (2-tailed)	.504	.030	.184	.317	.044				
	N	63	63	62	63	57	63			
RBDSQ	Coefficient	.148	-.086	-.213	-.069	-.111	-.032	--		
	Sig. (2-tailed)	.278	.528	.118	.614	.421	.813			
	N	56	56	55	56	55	56	56		
Hue test	Coefficient	-.024	.019	.098	.180	-.108	-.152	.071	--	
	Sig. (2-tailed)	.859	.889	.471	.179	.423	.260	.606		
	N	57	57	56	57	57	57	55	57	
Sniffin sticks	Coefficient	.174	-.087	.069	-.233	.056	.110	-.079	-.200	--
	Sig. (2-tailed)	.196	.518	.611	.081	.682	.416	.568	.135	
	N	57	57	56	57	57	57	55	57	57

**. Correlation is significant at the 0.01 level (2-tailed).

*. Correlation is significant at the 0.05 level (2-tailed).

Supplementary Table 3. Correlations between IL-2 and clinical data. Table shows the correlation coefficient and p value for Pearson correlation analysis between serum IL-2 and clinical data for iRBD patients. In some instances participants did not complete all clinical tests and this is reflected by lower sample sizes.

	Control	iRBD	*p* value
CD4	83.8 ± 1.5	86.0 ± 1.1	0.240
CD8	11.7 ± 1.1	9.9 ± 0.9	0.219
Th1	0.99 ± 0.3	1.22 ± 0.9	0.439
Treg	2.95 ± 0.20	2.99 ± 0.15	0.856

Supplementary Table 4. T cell subset frequency in study participants. Flow cytometry was used to assess T-cell populations in peripheral blood mononuclear cells from control (n=35) and iRBD (n=65) participants. The table shows the percentage ± standard error of T cell populations after univariate analysis covarying for age and sex.

	UPDRS-III	Serum IL-2
CD4+ IL2+	R = 0.11, p = 0.43	R = -0.16, p = 0.24
CD4+ IL4+	R = 0.17, p = 0.22	R = -0.06, p = 0.66
CD4+ IL-10+	R = 0.02, p = 0.89	R = 0.05, p = 0.71

Supplementary Table 5. Correlation of T cell subset frequency to UPDRS-III and serum IL-2 in iRBD participants. Flow cytometry was used to assess T-cell populations in peripheral blood mononuclear cells from iRBD participants (n=65) and the frequencies correlated to UPDRS-III and IL-2 using Pearson correlations. The table shows the correlation coefficient and p value.

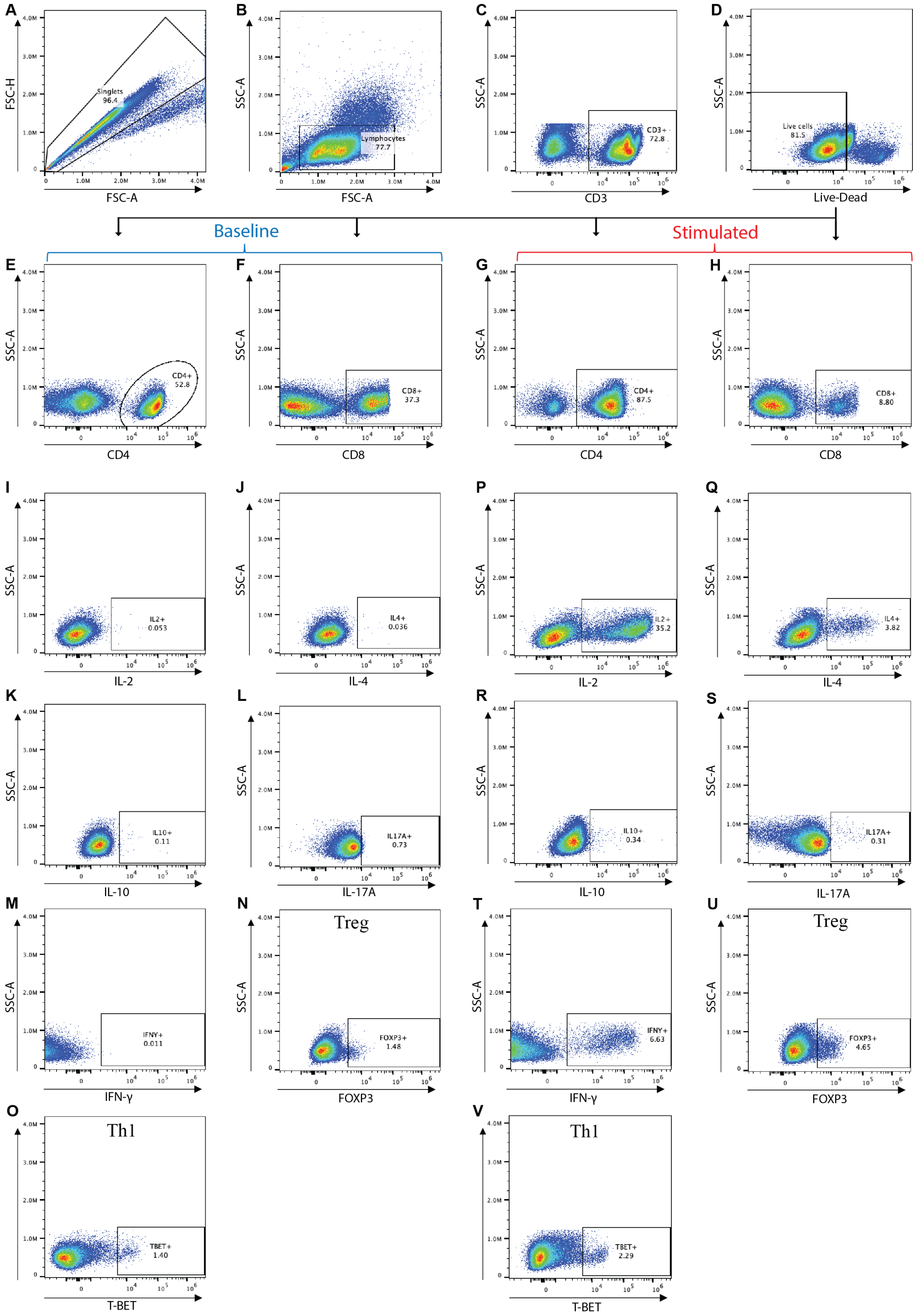

Supplementary figure 1. Representative flow cytometry gating strategy for identifying transcription factor and cytokine expression in CD4+ T cells.

Cryorecovered PBMC samples were stained for flow cytometry and data were acquired using a Cytek Aurora spectral analyser. Firstly, a singlet gate was created using FSC-A and FSC-H (A), followed by broad selection of lymphocytes based on size and granularity (B). Cells positive for CD3 were selected (C) followed by a live cell gate (D). Monocytes expressing CD4

or CD8 in unstimulated samples (E and F, respectively) and stimulated samples (G and H, respectively) were selected. In the CD4-positive T cell subset, expression of cytokines IL-2, IL-4, IL-10, IL-17A, IFN-γ and master transcriptional regulators FOXP3 and T-BET were quantified in unstimulated baseline samples (I-O) and in PMA/ionomycin stimulated samples (P-V).

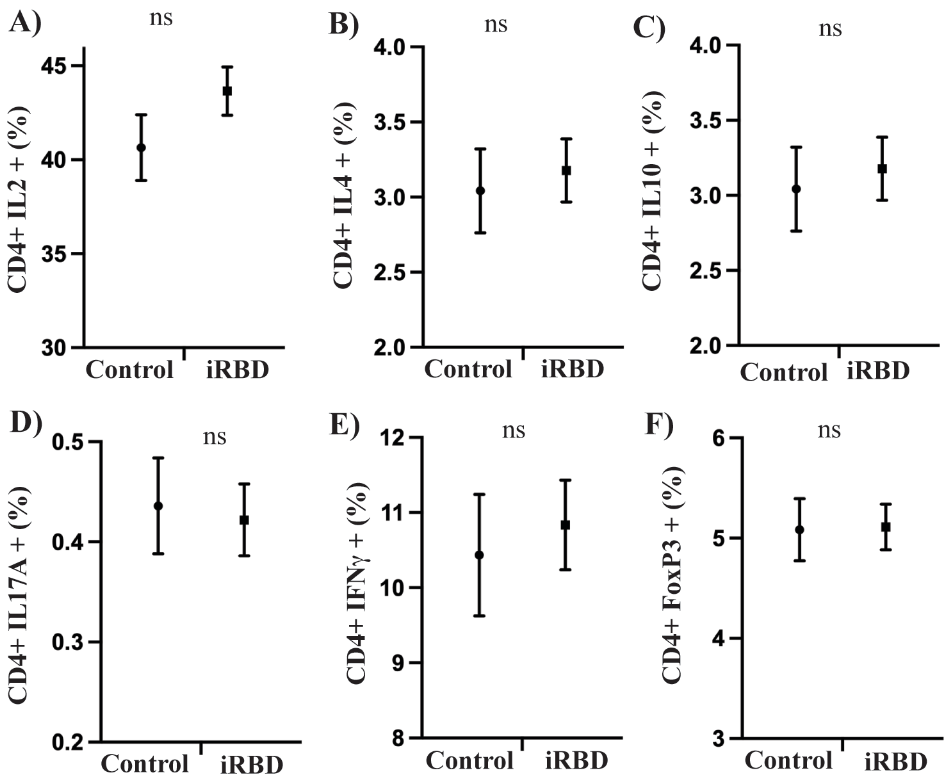

Supplementary Figure 2. Flow cytometry was used to assess the frequencies of activated CD4 T cells positive for IL-2 (**A**), IL-4 (**B**), IL-10 (**C**), IL-17A (**D**) and IFNγ (**E**). The graphs show the estimated marginal mean and the standard error of the mean following univariate analysis covarying for age and sex.
